# Comprehensive identification and analysis of circRNAs during hickory (*Carya cathayensis* Sarg.) flower bud differentiation

**DOI:** 10.3389/fpls.2022.1000489

**Published:** 2023-01-04

**Authors:** Hongmiao Jin, Zhengfu Yang, Jia Luo, Caiyun Li, Junhao Chen, Kean-Jin Lim, Zhengjia Wang

**Affiliations:** State Key Laboratory of Subtropical Silviculture, College of Forestry and Biotechnology, Zhejiang A&F University, Hangzhou, Zhejiang, China

**Keywords:** hickory, flowering, ceRNA network, circular RNA, circRNA

## Abstract

Flower bud differentiation represents a crucial transition from vegetative growth to reproductive development. *Carya cathayensis* (hickory) is an important economic species in China, with a long juvenile period that hinders its commercial development. In recent years, circular RNAs (circRNAs) have been widely studied and identified as sponges for miRNA regulation of mRNA expression. However, little is known regarding the role of circRNAs in flower buds. In this study, we sequenced circRNAs at three developmental stages (undifferentiated, differentiating, and fully differentiated) in both female and male buds. A total of 6,931 circRNAs were identified in the three developmental stages and 4,449 and 2,209 circRNAs were differentially expressed in female and male buds, respectively. Gene ontology demonstrated that many circRNA host genes participated in various processes, for example, cellular and intracellular pH regulation. Function annotation identified 46 differentially expressed circRNAs involved in flowering regulation, with 28 circRNAs found only in female buds, 4 found only in male buds, and 11 found in both female and male buds. A circRNA-miRNA-mRNA network was predicted based on 13 flowering-related circRNAs and their seven putative interacting miRNAs to describe the regulatory mechanism. Our preliminary results demonstrated a potential involvement of circRNA in bud differentiation. They provided a preliminary theoretical basis for how circRNA might participate in flower development in hickory, perhaps in woody plants.

## 1 Introduction

Hickory (*Carya cathayensis* Sarg.) is a woody plant species with prominent economic value for its nuts and oil. It is mainly distributed in the Tianmu Mountains, at the junction of Zhejiang and Anhui provinces (Yang et al., 2015). It is a monoecious tree with a long juvenile period and different developmental times for the male and female flowers. The female flower buds differentiate in mid-March and then develop into flower organs in mid-April. By contrast, vegetative growth of the male flower buds occurs in mid-late April, with differentiation completed in early May (Huang et al., 2006; Huang et al., 2007; Huang et al., 2013). This type of development is known as heterodichogamy, a mechanism that avoids inbreeding, and is common in 21 genera in 13 families, such as *Acer* in the Aceraceae and *Cyclocarya* and *Juglans* in the Juglandaceae (Kikuchi et al., 2009). However, this process results in low pollination, and therefore low fruit bearing rates, which hinders the hickory industry (Fukuhara and Tokumaru, 2014; Chen et al., 2019). Traditional breeding methods, such as natural variation selection and cross-breeding, have been applied to shorten the juvenile period and promote fruit-bearing. Nevertheless, hybrid breeding usually takes 5-10 years, and the effects are usually unsatisfactory. Therefore, understanding the regulatory mechanisms of male and female flower development at the molecular level might aid in solving some of these breeding problems.

The process of flowering has received intensive study. To date, the external and internal factors understood to regulate flower development fall into five major pathways associated with photoperiod, vernalization, autonomous, gibberellin (GA), and sucrose (Roldán et al., 1999; Mouradov et al., 2002). Transcription factors (TFs) have been reported to participate in at least two of these pathways and are called integrators of flowering regulation. In *Arabidopsis*, *FLOWERING LOCUS C (FLC)*, a central repressor of flowering first identified in the primordial cell, promotes the formation of the floral meristem (FM) and influences flowering by vernalization (Whittaker and Dean, 2017). *SHORT VEGETATIVE PHASE (SVP)*, which belongs to MADS-box family, interacts with *FLC* and binds to the CArG motif of the *FLOWERING LOCUS T (FT)* promoter to suppress *FT* expression (Jeong et al., 2007).

In addition to TFs, non-coding RNAs (ncRNAs) also play a role in flower regulation. For example, the vernalization-mediated epigenetic repression of *FLC* requires a long intronic noncoding RNA (lncRNA), COLD ASSISTED INTRONIC NONCODING RNA (COLDAIR) (Heo and Sung, 2011). Similarly, COOLAIR, another lncRNA identified in *Arabidopsis*, arises from the 3’ end of *FLC* in an antisense direction relative to *FLC* (Chekanova, 2015). Both lncRNAs repress the expression of *FLC via* an epigenetic mechanism to regulate flowering in *Arabidopsis* (Štorchová, 2017). *SVP* changes the expression of one of the ambient temperature-responsive miRNAs, micro RNA172 (miR172), and a subset of its target genes (Lee et al., 2010).

Research has shown that only 2% of the transcripts of a genome are translated into protein. A vast amount of the transcriptome is ncRNAs, including ribosomal RNAs (rRNAs), transfer RNAs (tRNAs), micro RNAs, short interfering RNAs (siRNAs), circular RNAs (circRNAs), and long-coding RNAs (Jandura and Krause, 2017). At present, functional studies of ncRNAs have mainly been conducted in the medical field, largely in cancer studies (Mattick and Makunin, 2006; Wang et al., 2014). Research on ncRNAs in plants has received relatively little attention until recently. Substantial research has now demonstrated that ncRNAs play an indispensable role in plant functions, including responses to abiotic stress, plant development, and fruit development (D’Ario et al., 2017; Correa et al., 2018; Tong et al., 2018; Waititu et al., 2020; Gelaw and Sanan-Mishra, 2021). For example, overexpression of osa-miR5506, a miRNA, resulted in pleiotropic abnormalities, including defects in ovary development, confirming a vital role for osa-miR5506 in regulating floral number and female gametophyte production (Chen et al., 2021). Similarly, circRNAs were revealed to act as miRNA sponges and to form competing endogenous RNA (ceRNA) networks to inhibit miRNA activities (Hansen et al., 2013; Zhang et al., 2017a).

CircRNAs were first discovered and experimentally verified in the 1970s. They were first considered a by-product of mistaken translation, but are now regarded as a class of covalently closed-loop RNAs characterized by their 3’ end and 5’ ends (Nigro et al., 1991). Four mechanisms are proposed to explain the generation of circRNA: back-splicing, intron-driven cyclization of complementary sequences, complementary cyclization driven by different introns from the same single gene, and exon cyclization regulated by RNA binding proteins (Sun et al., 2016). CircRNAs can be produced from exons, introns, or intergenic regions, but those produced from exons have received the most attention (Li et al., 2018).

The functions of circRNAs include transcriptional regulation, miRNA sponging, and translation into proteins. Some circRNAs may influence biological processes by regulating the expression of their parental genes (Zhou et al., 2020). For example, in *Arabidopsis*, circSEP3, which is derived from an exon of *SEPALLATA3*, regulates the splicing of its cognate mRNA by forming R-loop to affect floral development (Conn et al., 2017). In tea plants, the abundances of circRNAs were positively correlated with the mRNA transcript level of their parental genes and were considered to play a role in leaf development (Tong et al., 2018). In rice, overexpression of a linear Os08circ16564 construct reduced the expression of its parental gene in the leaf and panicle (Lu et al., 2015). By contrast, the circRNAs that act as sponges must possess rich miRNA binding sites or show high expression in the cytoplasm (Ashwal-Fluss et al., 2014). At present, only a few circRNAs from plants (5% in Arabidopsis and 6.6% in rice) have been demonstrated to contain miRNA binding sites (Ye et al., 2015). In Arabidopsis, circRNA biogenesis is altered by heat stress, leading to the suggestion that circRNAs may participate in heat stress responses through circRNA-mediated ceRNA networks. Recent research on rice has identified 11 circRNAs that were predicted to act as miRNA sponges that functioned in flag leaf senescence through the formation of circRNA-miRNA-mRNA ceRNA networks (Pan et al., 2018; Zhou et al., 2020; Huang et al., 2021). In *Brassica campestris*, a ceRNA and miRNA-mRNA network containing the circRNA A02:23507399|23531438 was hypothesized to act as a miRNA sponge for the mRNAs unconservative_A06_21945 and unconservative_Scaffold000096_42992 to regulate the expression of Bra002275 and the biosynthesis of tryphine and sporopollenin (Liang et al., 2019).

In the present study, we explored the role of circRNAs in regulating hickory flower development by examining six circRNA libraries obtained from the transcriptome of female and male flowers. At different developmental stages, we observed different circRNA expression profiles. After annotating and predicting the target miRNAs, we proposed possible circRNA-miRNA-mRNA regulatory networks that could be involved in hickory flower development. Our preliminary results shed light on the potential role of circRNAs in hickory flower bud development.

## 2 Materials and methods

### 2.1 Plant materials and sample collection

Male and female flower buds were collected from 15-year-old asexually propagated hickory trees growing in the nursery orchard of Zhejiang A&F University (lat. 30°15’N, long. 119°43’E), Zhejiang Province, China. Female flower buds were collected at the F1 undifferentiated stage (early March, 2016), the F2 differentiating stage (late March, 2016), the F3 fully differentiation stage (April, 2016). Male flower buds were collected at the M1 undifferentiated stage (April, 2016), the M2 differentiating stage (May, 2016) and the M3 fully differentiation stage (June, 2016) (Huang et al., 2006; Huang et al., 2007; Huang et al., 2013). All samples were immediately immersed in liquid nitrogen and stored at -80 °C until RNA extraction.

### 2.2 Total RNA extraction, library construction, and sequencing

Total RNA was isolated from female and male hickory buds at different developmental stages using a modified CTAB method (Lim et al., 2016) combined with TRIzol reagent (Invitrogen, Grand Island, NY, USA). A total of 3 μg of total RNA per sample was used as the starting material for the RNA sequencing (RNA-seq) libraries preparation. Ribosomal RNA depletion and RNA-seq library preparation were performed as described in Li and colleagues (Li et al., 2022).

A total of six libraries were sequenced on an Illumina Hiseq 2500 platform. After sequencing, the low-quality and adapters sequences were removed from the raw data using NGS QC Toolkit 2.3.3 software (Patel and Jain, 2012) to obtain clean data.

### 2.3 Identification of circRNAs

For circRNA recognition, find_circ and CIRI2 software were used to identify circRNA from female and male flower buds (Memczak et al., 2013; Gao et al., 2018). The find_circ utilized bowtie2 reference matching to extract 20-nt anchor sequences as a seed sequence from each read end that match the reference sequence. Each pair of anchor sequences was compared to the reference sequence again. If the 5’ end of the anchor sequence matched the reference sequence (A3 and A4 for the start and end sites, respectively), and the 3’ end of the anchor sequence matched upstream of that site (A1 and A2 for the start and end sites, respectively), and a splice site was present between A2 and A3, then that read was identified as a candidate circRNA. The candidate circRNA with a read count greater than or equal to two was used as the identified circRNA. CIRI2 searched for a paired chiastic clipping (PCC) signal, and a paired end mapping (PEM) signal, with a GT-AG signal first. It then filtered the candidate circRNAs based on the global comparison, the reads support of circRNAs, and the annotation information to identify junction reads. Those with a read number larger than two were selected as the identified circRNA (Gao et al., 2015; Ye et al., 2015; Tong et al., 2018; Zhang et al., 2019; Li et al., 2020; Philips et al., 2020; Yang et al., 2020; Jiang et al., 2021). The results that appeared in both find_circ and CIRI2 were selected as candidate circRNAs (Xu et al., 2018; Liu et al., 2019; Li et al., 2020; Philips et al., 2020; Yang et al., 2020).

### 2.4 Bayesian hierarchical clustering analysis

The newly identified circRNAs were mapped against the hickory genome (Huang et al., 2019), and the mapped count tables of female and male flower bud libraries were obtained using featureCounts software (version 1.20.6, Liao et al., 2014). The mapped count tables were then loaded into the edgeR (version 3.2.4, Robinson et al., 2009) R session (version 3.5.0). The raw counts were normalized using the trimmed mean of M-value method to obtain normalized counts per million (CPM). The average CPM and relative mean CPM counts were calculated, and the expression level of circRNA was set to 1, as described by Lim et al. (2021). Bayesian hierarchical clustering analysis was carried out using Spline Cluster (version 2002, Heard et al., 2006), with the default parameters, except that prior precision and normalize targets were set to 1 e-45 and 0, respectively. Clusters were visualized using SplineCluster (Lim et al., 2021).

### 2.5 Functional enrichment analysis

An expression level of circRNA greater than or equal to two CPM was defined as differential expression in both female and male flower bud data sets (Singh et al., 2014). The differently expressed (DE) circRNAs of female and male flower buds were subjected to subsequent analysis. We performed GO enrichment analysis for the host genes of DE circRNAs to better understand how circRNA may be involved in flower development. GOseq (version 2.12) was used to annotate the function of the parent genes of the differentially expressed circRNAs’ host genes (Young et al., 2010) using the Wallenius non-central hyper-geometric distribution method (Kang and Liu, 2015; Bedre et al., 2019; Lipka et al., 2019; Li et al., 2021). The Benjamini Hochberg method was used to correct the p-value, with a smaller value being more significant. To further understand how circRNAs may be involved in bud differentiation, we proposed a regulatory network of flowering-related genes and combined the expression profiles of circRNAs characterized by different expressions in the three differentiation stages.

### 2.6 Prediction of target miRNAs site

To explore the regulatory role circRNAs might play as ceRNAs, the functions circRNA in bud differentiation were examined by predicting the target miRNAs of differentially expressed circRNAs in undifferentiated and differentiated stages and the corresponding mRNAs of miRNA. The miRNA binding sites of the circRNA were predicted using miRanda (version 3.3a, Enright et al., 2003). The predicted results of miRNA binding sites were used to deduce the circRNA-miRNA regulatory networks using Cytoscape software (version 3.8.2) with default parameters (Shannon et al., 2003).

### 2.7 Real-time quantitative RT-PCR (qRT-PCR) analysis

The female hickory flower buds corresponding to undifferentiated, differentiating, and fully differentiated stages were collected in early March, late March, and April, 2021, respectively. The male flower buds at the same differentiation stages were collected in April, May, and June 2021, respectively. Total RNA was isolated as described above. A total of nine flowering-related circRNAs were selected from the differentially expressed circRNAs for RNA-seq verification. The hickory histone sequence was used as an internal reference gene. The relative expression level was calculated using the 2^−ΔCt^ method (de Vos et al., 2017). All primers were designed using primer3plus (https://primer3plus.com/) and were listed in **Supplementary Table S13**.

## 3 Results

### 3.1 Identification of circRNAs in female and male hickory flower buds

The regulatory mechanism of circRNA during hickory flower bud differentiation was revealed using RNA-sequencing (RNA-seq) to mine circRNAs in female (F) and male (M) flower buds at different developmental stages. All the acquired transcriptomes were divided into six libraries: F1 and M1 for the undifferentiated stage, F2 and M2 for the differentiating stage, and F3 and M3 for fully differentiated stage. Mapping to the hickory genome, as shown in **Supplementary Table S1**, results showed that, averagely the male flower bud libraries had a higher unique mapping rate than the female flower bud libraries.

Between the three developmental stages, an average of 7,472 and 4,381 circRNAs were identified in female and male flower buds. Among all the identified candidate circRNAs, 83.63% in the female buds and 84.19% in the male buds were from exons (**Supplementary Table S3**). Our results showed that most of the candidate circRNAs were from exons, with only about 5% derived from introns in both female and male flower buds (**Supplementary Figure S1A**). We noticed that over 85% of the circRNAs were shorter than 5000 nt, and nearly 1% were between 5000 nt and 10000 nt in length. The number of circRNAs shorter than 500 nt peaked in all six circRNA libraries. In general, 60.34% of circRNAs were less than 1500 nt in length (**Supplementary Figure S1B**).

**Figure 1:**
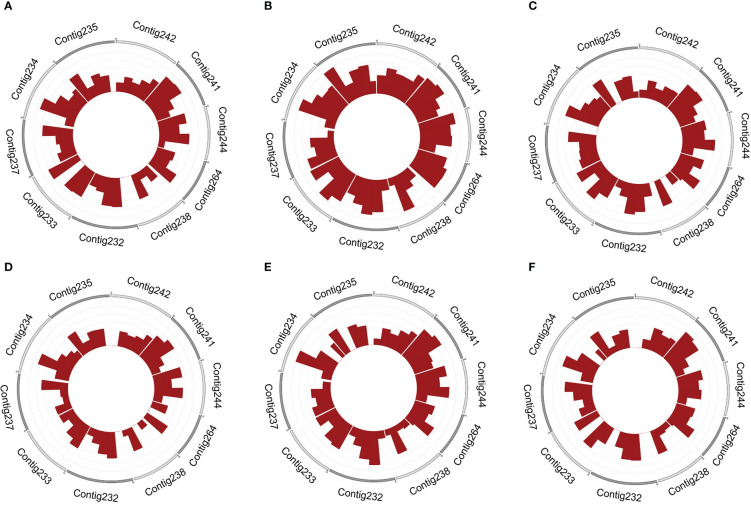
The density of circRNAs in female and male flower buds on different contigs. The 10 most densified distributed contigs in female and **(A–C)** and male **(D–F)** flower buds, respectively. The outer ring takes 10 contigs for display. A contig with more rings has a higher density of circRNAs.

Chromosomal localization analysis of the candidate circRNA showed that contig232, contig233, contig234, contig241, contig244, and contig264 transcribed the largest number of circRNAs in female buds. By contrast, the largest number of circRNAs transcribed in male buds were in contig232, contig233, contig234, contig237, contig241, and contig244 (**Figure 1**).

### 3.2 The expression profiles of circRNAs in developing hickory flowers

A total of 6,931 circRNAs were identified (**Supplementary Figure S2**). The 6,172 circRNAs in female buds and 3,349 circRNAs in male buds contained mapped counts. Further analysis of these circRNAs demonstrated the expression of 2,336 circRNAs in F1, 5,145 in F2, and 2,280 in F3 during the development of female flower buds. Of these, 484 circRNAs were only expressed in F1, while 636 circRNAs participated in the development process of female flower buds. During the development of male flower buds, a total of 1,434 circRNAs were expressed during the M1 stage, 2,229 circRNAs were involved in the flower bud differentiation process during the M2 stage, and 1854 circRNAs were expressed during the M3 stage. A total of 697 circRNAs were involved in the whole progress of male flower bud differentiation and development (**Supplementary Tables S4**, **S5**).

**Figure 2:**
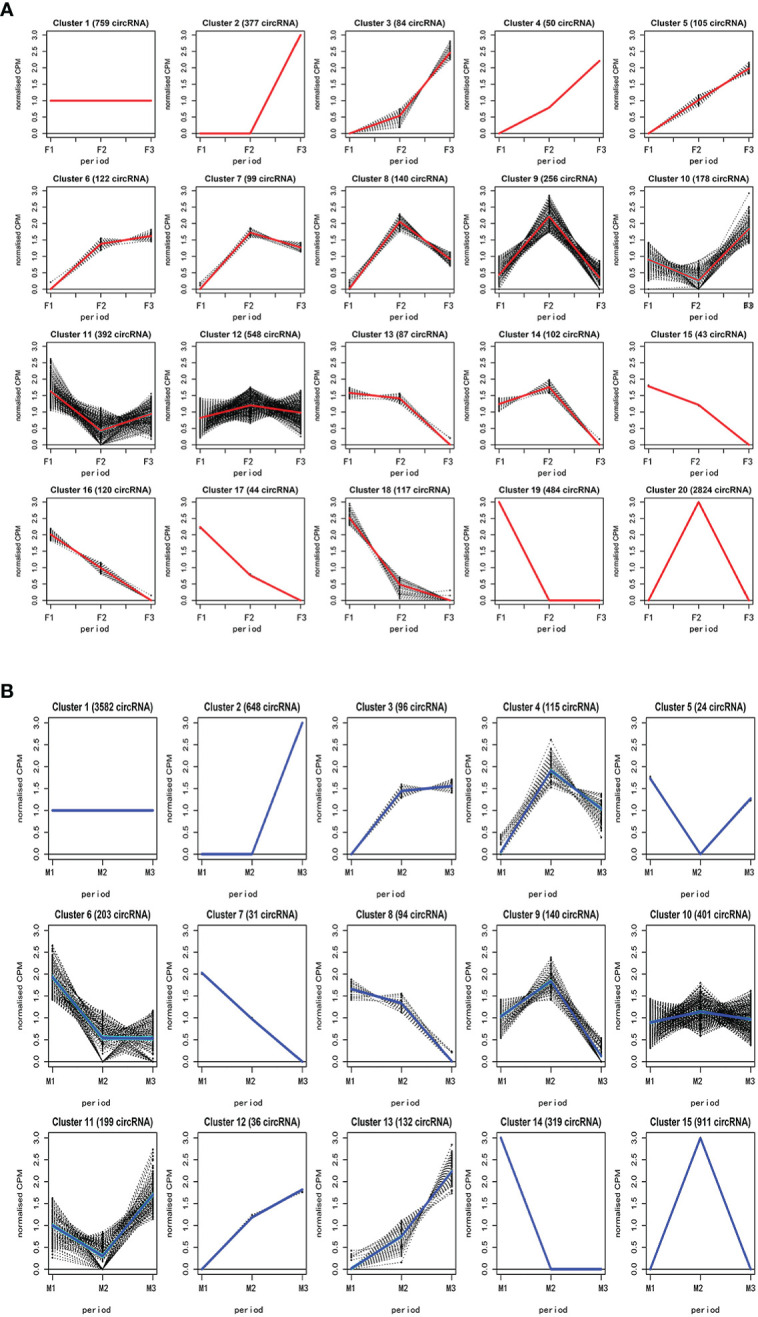
The hierarchical clustering analysis of circRNAs in female and male buds. The circRNAs in female hickory flower buds were divided into 20 clusters **(A)** while the circRNAs of male flower buds were grouped into 15 clusters **(B)**. The analysis was performed with the Bayesian approach to reveal the expression profile of circRNAs in female and male bud using relative mean counts per million (CPM) counts. F, female; M, male; F1 and M1, undifferentiated stage; F2 and M2, differentiating stage; F3 and M3, fully differentiated stage.

We performed clustering analysis with the Bayesian approach to reveal the expression profile of 6,931 circRNA of female and male flower buds using CPM counts. The clustering analysis showed that circRNAs in female flower buds were divided into 20 clusters (**Figure 2A**) with several expression profiles. In the first profile, in clusters 2-6, the expression was a constitutively increasing expression from F1 to F3, whereas clusters 7-9, 12, 14, and 20 showed peak expression in F2. By contrast, clusters 10 and 11 had their lowest expression in F2. CircRNAs in clusters 13 and 15-19 were consistently downregulated. Only circRNAs in cluster 1 had a relatively constant expression profile, and they may be involved in maintaining basic biological functions. The 4,449 differentially expressed circRNAs (CPM >2) in female flower buds were distributed in clusters 2-4 and 17-20 (**Supplementary Table S6**).

Clustering analysis also grouped the circRNAs of male flower buds into 15 clusters (**Figure 2B**).Upregulation profiles across all stages were observed in clusters 2-3 and 12-13, whereas clusters 4, 9-10, and 15 shared a similar profile, with the peak expression at the M2 stage, and clusters 5 and 1 shared a similar profile with peak expression at M2. Clusters 5 and11 had the opposite profile, showing the lowest expression at M2. Clusters 6-8 and 14 shared the downregulation profiles. The circRNA in cluster 1 shared the same profile as that in the female flower. A total of 2,209 circRNAs (CPM >2) were differentially expressed during male bud development and were distributed in clusters 2, 7, and 13-15 (**Supplementary Table S7**).

### 3.3 Enrichment analysis of differentially expressed circRNA host genes

Female flower bud circRNAs in cluster 2 were enriched with biological process terms including 1,3-beta-D-glucan synthase complex (GO:0000148), 1,3-beta-D-glucan synthase activity (GO:0003834), (1->3)-beta-D-glucan metabolic process (GO:0006074), and (1->3)-beta-D-glucan biosynthetic process (GO: 0006075). Positive regulation of the biological process (GO:0048518) was also enriched. CircRNAs in cluster 4 were enriched by many molecular function terms, including thiol-dependent ubiquitin-specific protease activity (GO:0004843), ubiquitin-like protein-specific protease activity (GO:0019783), cysteine-type peptidase activity (GO:0008234), and serine-type endopeptidase activity (GO:0004252). Several biological processes, such as protein phosphorylation (GO:0006468) and peptidyl-serine modification (GO:00182095), were enriched in cluster 17. Terms enriched in cluster 19 were diverse and included purine nucleotide binding (GO:0001883), guanyl nucleotide binding (GO:0019001), and mRNA metabolic processes (GO:0016071). Terms in cluster 20 were more complex (**Supplementary Table S8**).

In male flower buds, cellular components, such as vacuolar membrane (GO:0005774), vacuolar part (GO:0044437), and vacuolar organization (GO:0007033), were enriched in cluster 2. Terms concerning beta-D-glucan, such as GO:0000148, GO:0003834, GO:0006074, and GO:0006075, were also enriched. Interestingly, phosphate-related terms, including positive regulation of phosphorylation (GO: 0042327 and positive regulation of phosphate metabolic processes (GO: 0045937), were enriched in cluster 2. Cluster 13 was enriched with ubiquitin-related terms, such as ubiquitin-like protein-specific protease activity (GO: 0019783), thiol-dependent ubiquitinyl hydrolase activity (GO:0036459), and ubiquitinyl hydrolase activity (GO:0101005). In cluster 15, we noted that regulation of cellular pH (GO:0030641) and regulation of intracellular pH (GO:0051453) were enriched (**Supplementary Table S9**).

### 3.4 Functional analysis of differentially expressed circRNAs

**Figure 3:**
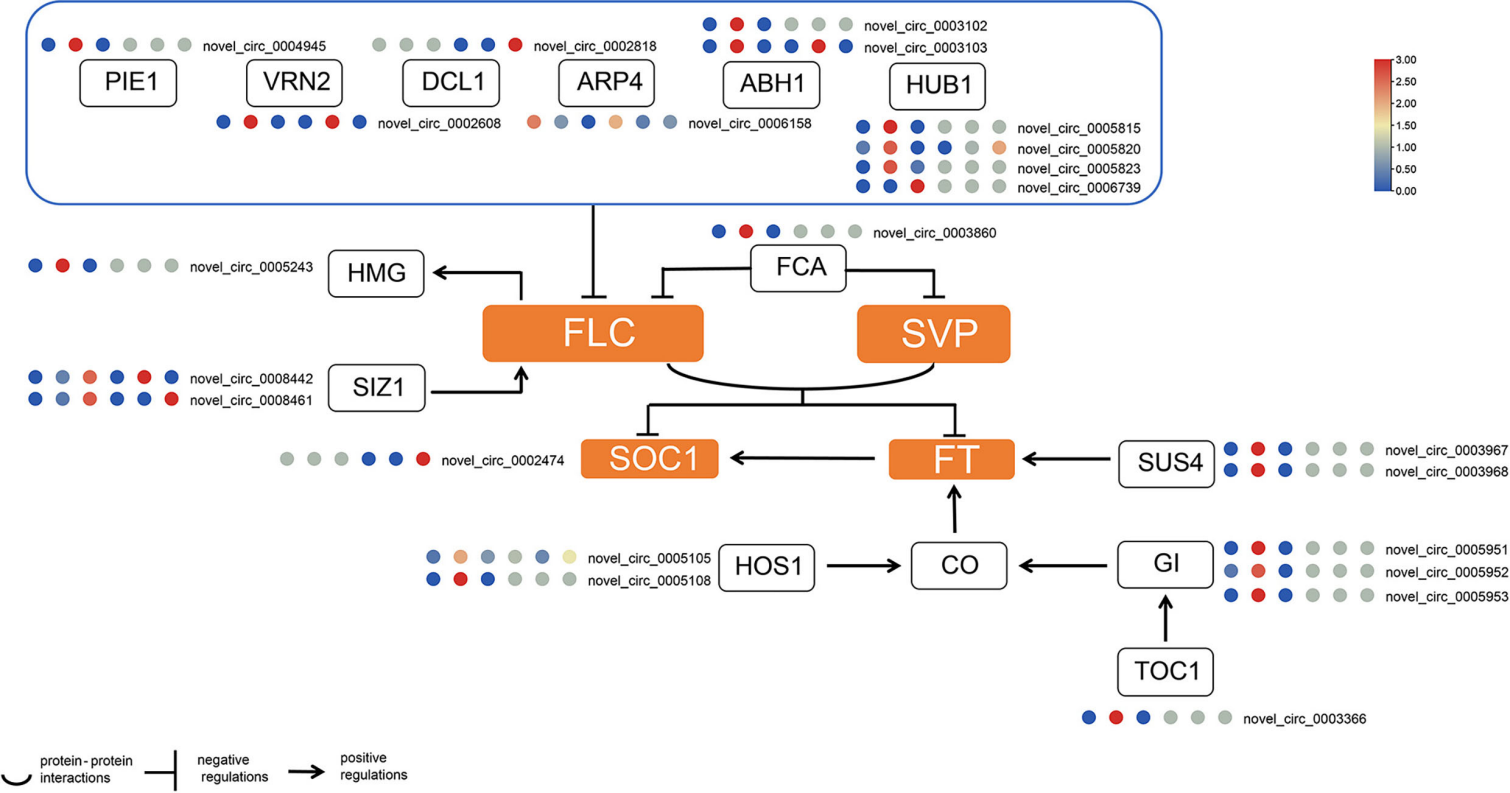
Transcript abundance of flowering-related circRNAs in hickory buds. The circRNA expression level was presented in relative mean counts per million (CPM). The six plots from leftmost to the rightmost represent F1, F2, F3, M1, M2 and M3 respectively. The scale bar is the relative mean of CPM. The square denotes genes or proteins, while the orange represents integrator gens or proteins. *PEI1*, *PHOTOPERIOD-INDEPENDENT EARLY FLOWERING 1*; *HUB1*, *HISTONE MONOUBIQUITINATION1*; *VRN2*, *VERNALIZATION 2*; *DCL1*, *DICER-LIKE 1*; *ABH1*, *ABA HYPERSENSITIVE 1*; *ARP4*, *ACTIN-RELATED PROTEIN 4; HMG*, *HIGH MOBILITY GROUP*; *FCA*, *FLOWERING CONTROL LOCUS A*; *SIZ1*, *SALT INDUCED ZINC FINGER PROTEIN1*; *FLC*, *FLOWERING LOCUS C*; *SVP*, *SHORT VEGETATIVE PHASE*; *SOC1*, *SUPPRESSOR OF OVEREXPRESSION OF CO 1*; *FT*, *FLOWERING LOCUST T*; *SUS4*, *SUCROSE SYNTHASE 4*; *HOS1*, *HIGH EXPRESSION OF OSMOTICALLY RESPONSIVE GENES 1*; *CO*,*CONSTANS*; *GI*, *GIGANTEA*; *TOC1*,*TIMING OF CAB EXPRESSION 1*.

Due to the imperfection of the reference genome, a large number of circRNAs were not annotated. Therefore, we first performed a functional homology search on the obtained circRNAs that were not annotated in the genome. The results identified a total of 46 circRNAs that may be involved in female and male flowering processes. In general, more circRNAs involved in flowering were identified in female flowers; four were specifically expressed in male buds and 11 were expressed in both female and male buds (**Supplementary Table S10**). Our results showed that certain circRNAs were derived from *MADS-box* genes. Novel_circ_0002474 was predicted to be derived from *SUPPRESSOR OF OVEREXPRESSION OF CO 1 (SOC1)*, and novel_circ_0005951, novel_circ_0005952 together with novel_circ_0005953 were thought to be products of *GIGANTEA (GI)*. Novel_circ_0004945 was from a host gene encoding the PHOTOPERIOD-INDEPENDENT EARLY FLOWERING 1 (PIE1) protein. Four circRNAs were derived from *HISTONE MONOUBIQUITINATION1* (*HUB1)*; only novel_circ_0005820 was found in both female and male buds. Novel_circ_0005815 and novel_circ_0005823 were from the same parental gene, *HUB1*. Their similar expression profiles suggested that they may be functionally redundant. The *GI*-related circRNAs were novel_circ_0005951, novel_circ_0005952, and novel_circ_5953, and all three circRNAs were differentially expressed in F1 and F2 in the female buds. Their expression profiles hint that they may be functionally redundant. Novel_circ_0008461 and novel_circ_0008442 were from genes encoding SALT INDUCED ZINC FINGER PROTEIN1 (SIZ1), Novel_circ_0008461 had significantly rich expression in the third stage in flower buds (**Figure 3**).

### 3.5 Interaction of differentially expressed circRNA-miRNA-mRNA networks

**Figure 4:**
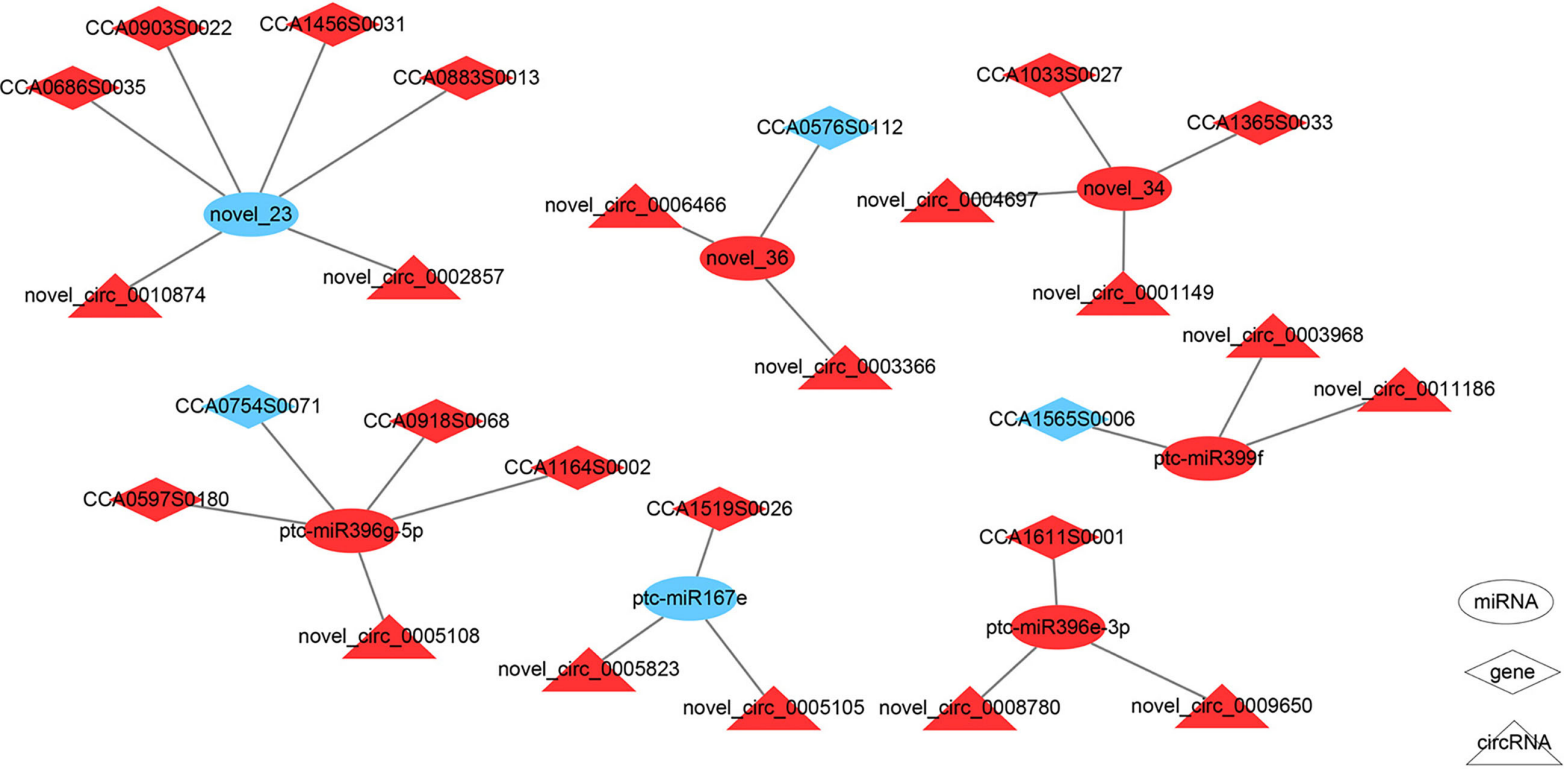
The circRNA-miRNA-mRNA regulation network in F1 compared with F2. The ellipses, diamonds, and triangles denote miRNAs, genes, and circRNAs, respectively. The red denotes upregulation and the blue denotes downregulation.

Studies have shown that circRNAs can perform regulatory functions by adsorbing specific miRNAs that function in multiple life processes (Olesen and Kristensen, 2021). We identified 1,397 differentially expressed circRNAs targeting 17 miRNAs and regulating 22 mRNAs in the F1 compared with the F2 stages. By contrast, comparison of the M1 and M2 stages revealed 53 differentially expressed circRNA targeting two miRNAs with two mRNAs. The process was more complex in female buds, as 135 circRNAs that interacted with miR169i and miR169r were only present in female buds. (Lee et al., 2010) We noted that 127 circRNAs interacted with pct-miR399f, and 235 circRNAs interacted with pct-miR396e-3p; these are involved in plant development and were found only in female buds (**Supplementary Tables S11**, **12**). (Liebsch and Palatnik, 2020)Our regulatory network (**Figure 4**) using the differentially expressed circRNA and targeted miRNAs predicted that the axes novel_circ_novel_0002857-novel_23-CCA0686S0035/CCA1456S0031/CCA0883S0013/CCA0903S0022 presented up-down-up trends, suggesting that novel_circ_0002857 may act as the ceRNA of novel_23. Similarly, novel_circ_0010874 may have the same function as novel_circ_0002857, although they are derived from different genes. Both novel_circ_0005823 and novel_circ_0005105 have binding sites for ptc-miR167e targeting CCA1519S0026, and the expression profile indicated a putative sponge role for these two circRNAs.

### 3.6 Real-time quantitative RT-PCR (qRT-PCR)

**Figure 5:**
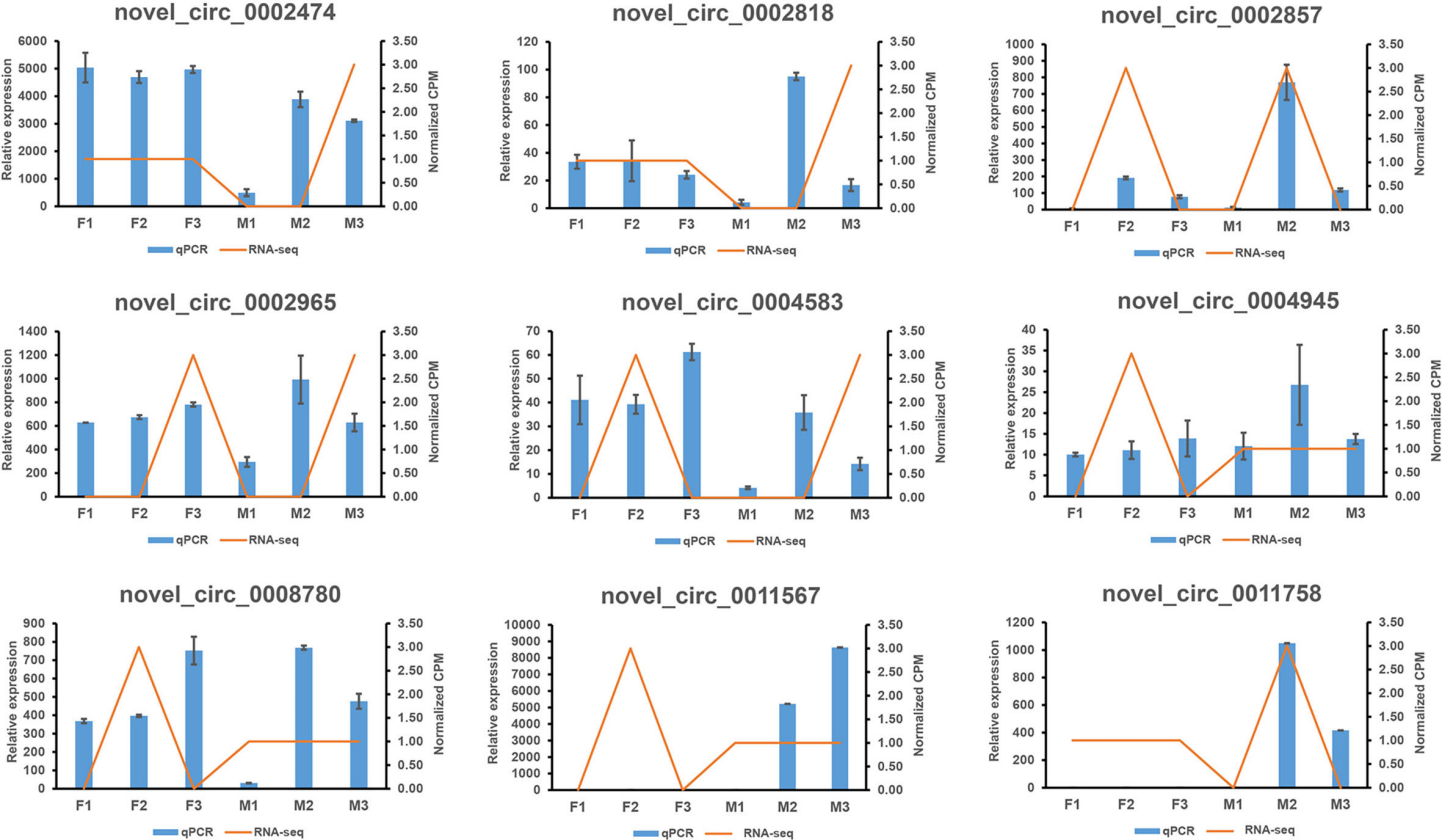
The comparison of real-time quantitative RT-PCR (qRT-PCR) and RNA sequencing (RNA-seq) for 9 circRNAs. The two results were in good agreement. The blue columns represented the results of the q RT-PCR, and the orange lines denoted for the results of the RNA-seq.

We randomly selected nine circRNAs and compared their expressions to those determined by RNA-seq. The qRT-PCR results were in good agreement with the RNA-seq results (**Figure 5**, **Supplementary Table 14**).

## 4 Discussion

### 4.1 Identification and characterization of circRNAs in hickory floral development

In this study, we identified 6,931 circRNAs in hickory flowers by RNA-seq. Many circRNAs have been observed in other plant species, such as *Arabidopsis thaliana* (6,012), *Oryza sativa* (12,307), and *Zea mays* (1,199) (Ye et al., 2015; Han et al., 2020). Our characterization of the circRNAs revealed more circRNAs in female buds. In both female and male buds, circRNAs from exons had a more prominent role, as observed in many other plants (Wang et al., 2022), suggesting that exonic circRNAs may serve as regulators in cells (Li et al., 2015). Of the differentially expressed circRNAs, 3,538 were explicitly expressed in female buds, while 1,298 circRNAs were only expressed in male buds. Female and male buds shared 911 expressed circRNAs. These results support that circular RNAs in female shoots might be involved in more diverse biological processes.

### 4.2 Functional analysis of differently expressed circRNA

The parental genes of circRNAs in clusters 2 to 4 in female buds generally presented an upregulation trend. Interestingly, the most enriched GO terms were positive regulators of biological processes (GO:0048518), suggesting that these circRNAs might contribute to cell development (Ishikawa et al., 1995). Cluster 20 showed peak expression in F2, with enrichment terms of ATPase activity (GO:0016887), nuclear transport (GO:0051169), enzyme binding (GO:0019899), and protein import (GO:0017038). The circRNAs generated from these parental genes may be involved in cell growth (Du et al., 2015). In male flower buds, members of cluster 2 showed expression beginning in M2 and were enriched with a batch of glucan-related GO terms, including 1,3-beta-D-glucan synthase complex (GO:0000148), 1,3-beta-D-glucan synthase activity (GO:0003834), (1->3)-beta-D-glucan metabolic process (GO:0006074), and (1->3)-beta-D-glucan biosynthetic process (GO:0006075). The results indicated possible activation of wall assembly by circRNAs (Yoshimi et al., 2017). Furthermore, members in cluster 15 had high expression in M2 but were silent in M1 and M3. We noted that regulation of cellular pH (GO:0030641) and regulation of intracellular pH (GO:0051453) were enriched in this cluster. Intracellular pH control is vital for many cellular behaviors, such as enzyme activity, protein degradation, and organelle activities (Ishaque and Al-Rubeai, 1998).

We traced circRNAs back to the parental genes and found that one parental gene can generate multiple circRNAs that can exhibit the same or different expression profiles. In this study, we noted that *SUS4*, *GI*, *HUA1*, and *HOS1* generated several circRNAs, while *HUB1* produced four circRNAs, with different expression profiles. Novel_circ_0005815 and novel_0005823 peaked in F2, while novel_circ_0006739 and novel_circ_0005820 peaked in F3. The different expression profiles indicated that they might have different functions (Conn et al., 2017).

### 4.3 The circRNA-miRNA-mRNA network in hickory floral development

CircRNA has been identified as a member of ceRNAs due to its abundance of conserved miRNA binding sites or miRNA response elements (MREs). Studies have illustrated that circRNA may act as ceRNAs (Cortés-López and Miura, 2016; Zhang et al., 2017b; Zhong et al., 2018). The ceRNA hypothesis proposes that RNAs sharing multiple MREs could have efficient crosstalk, forming a large-scale network in the transcriptome (Salmena et al., 2011). Therefore, we predicted the occurrence of different interactions among circRNA-miRNA-mRNA in F1 than in F2 and in M1 compared with M2. Among all the differentially expressed flower-related circRNAs, we only obtained 13 circRNAs, seven miRNA targets, and 14 mRNAs, all in F1 and F2. In our study, the expression level of circRNAs, such as novel_circ_0002857, novel_circ_0005105, and novel_circ_0005823, increased during female bud development, while their target miRNAs decreased (**Figure 4**, **Supplementary Tables S11** and **S12**), thereby leading to the promotion of targeted mRNAs. In brief, these circRNAs might act as miRNA sponges to regulate the expression of target mRNAs. The targeted miRNAs include miR169, miR396 and miR399 all were thought to play a role in flowering regulation (Lee et al., 2010; Xu et al., 2016; Liebsch and Palatnik, 2020). Recent research has demonstrated that these correlations may result from co-expression or mutual exclusivity in subpopulations in complex tissues; therefore, the expression profiles need confirmation (Zhang et al., 2019).

The results of expression profile analysis, host gene annotation, and circRNA-miRNA-mRNA prediction indicate that, among all four circRNAs derived from *HUB1*, only novel_circ_0005823 was predicted to have a miRNA binding site. Novel_circ_0005815 was derived from cluster 20 and had the highest expression among the four circRNAs, while novel_circ_0005823 was from cluster 9. Hence, we hypothesized that these circRNAs might play roles in different mechanisms. The other putative sponge of miR167 was novel_circ_0005105, which was grouped in cluster 9, while its sibling, novel_circ_0005108, was clustered in cluster 20 and was predicted to have a pct-miR396g-5p binding site. The target of miR167 was CCA1519S0026, annotated as a putative FT-like gene by NCBI. We hypothesized that novel_circ_0005823 and novrl_circ_0005105 may act as sponges for miR167, thereby contributing to the abundance of CCA1519S0026. We proposed that novel_circ_0003968 and novel_circ_0011186 promote the expression of ptc-miR399, which regulates LNC_02115 in the female hickory bud during temperature changes (Li et al., 2022). Nevertheless, more detailed mechanisms may also exist that need further experimental exploration.

## 5 Conclusions

We collected and constructed transcriptome datasets of female and male flower buds at three different developmental stages. From the transcriptome datasets, we identified 6,931 circRNAs in hickory buds. Characterization analysis indicated that most circRNAs were derived from exons and were less than 5000 nt in length. Expression analysis revealed that most circRNAs were expressed in female buds. In total, 4,449 and 2,209 differentially expressed circRNAs were identified in female and male buds, respectively. We studied the flowering-related candidates from the differentially expressed circRNA host genes and predicted the miRNA binding sites. Based on the ceRNA theory, we noted that novel_circ_0005823 and novel_circ_0005105 might target miR167 and regulate CCA15190026 to influence flowering. We also observed that novel_circ_0002857 and novel_circ_0010874 might serve as miRNA sponges of novel_23, thereby influencing flowering. Our preliminary results shed light on how circRNA might involve in the hickory flower development, perhaps in woody plants.

## Data availability statement

The original contributions presented in the study are publicly available. This data can be found here: NCBI, PRJNA820165.

## Author contributions

JL, ZW, and K-JL conceived and designed this study. HJ, JL, JC, and K-JL performed research and analyzed data. HJ and JL wrote the manuscript. K-JL and ZY edited and reviewed the writing. ZW and ZY acquired funding. All authors have read and agreed to the published version of the manuscript.

## Supplementary material

Supplementary Table 1 Linear alignment of reference genome.

Supplementary Table 2 Identification of circRNA in female and male buds.

Supplementary Table 3 The genomic feature of identified circRNAs.

Supplementary Table 4 The expression of circRNAs in female buds.

Supplementary Table 5 The expression of circRNAs in male buds.

Supplementary Table 6 The clusters of circRNAs in female buds.

Supplementary Table 7 The clusters of circRNAs in male buds.

Supplementary Table 8 Gene ontology analysis in female buds.

Supplementary Table 9 Gene ontology analysis in male buds.

Supplementary Table 10 Flower-related differentially expressed circRNAs.

Supplementary Table 11 The prediction of circRNA-miRNA-mRNA in F1 compared with F2.

Supplementary Table 12 The prediction of circRNA-miRNA-mRNA in M1 compared with M2.

Supplementary Table 13 Primers for qRT-PCR.

Supplementary Table 14 The original data and calculation of qRT-PCR.

Supplementary Figure 1 Characterizations of circRNA in female and male flower buds. **(A)** The distribution of circRNAs. The circRNAs derived from exons were indicated in red, and those from intergenic and introns were shown in green and blue, respectively. **(B)** The circRNA length distribution of six libraries. The female (F) and male (M) bud samples were collected at undifferentiated (F1, M1), differentiation (F2, M2), and differentiation completed (F3, M3) stages.

Supplementary Figure 2 The flowchart of circRNA identification and analysis.
