# Supplementary figures and images for: Comprehensive identification and analysis of circRNAs during hickory (Carya cathayensis Sarg.) flower bud differentiation
Source: Front Plant Sci. 2023 Jan 4;13:1000489. doi: 10.3389/fpls.2022.1000489 (PMC9846342; doi:10.3389/fpls.2022.1000489)

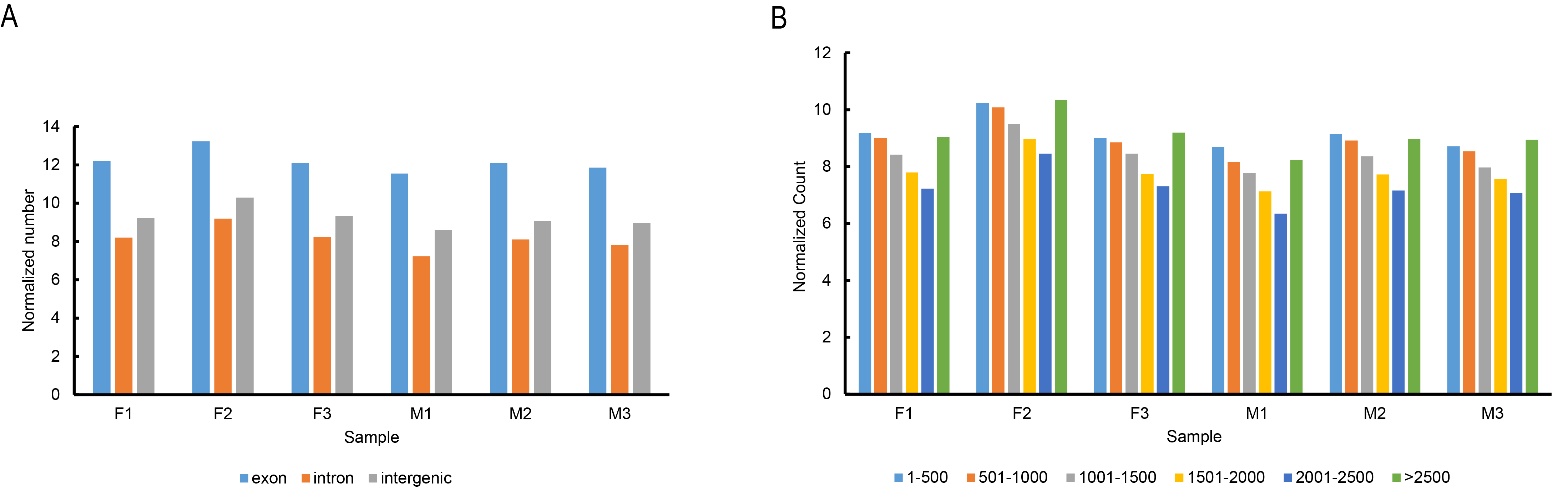

Supplement: Supplementary Figure 1 — Characterizations of circRNA in female and male flower buds. (A) The distribution of circRNAs. The circRNAs derived from exons were indicated in red, and those from intergenic and introns were shown in green and blue, respectively. (B) The circRNA length distribution of six libraries. The female (F) and male (M) bud samples were collected at undifferentiated (F1, M1), differentiation (F2, M2), and differentiation completed (F3, M3) stages. [file Image_1.jpeg]

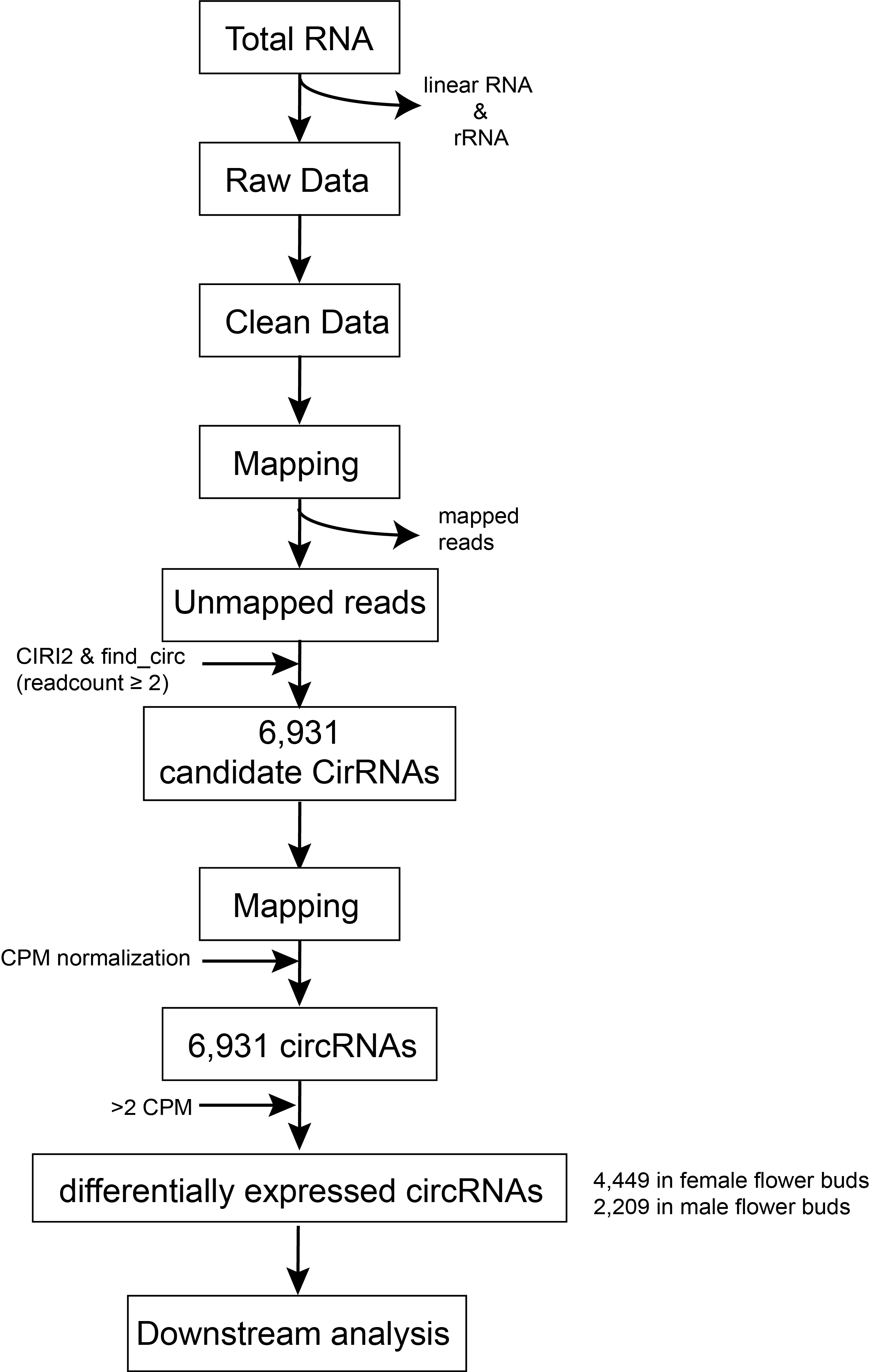

Supplement: Supplementary Figure 2 — The flowchart of circRNA identification and analysis. [file Image_2.jpeg]
